# The Dimensionality of the Multidimensional Fatigue Inventory (MFI-20) Derived From Healthy Adults and Patient Subpopulations: A Challenge for Clinicians

**DOI:** 10.7759/cureus.26344

**Published:** 2022-06-26

**Authors:** Daphne Bakalidou, Georgios Krommydas, Triantafyllia Abdimioti, Panagiotis Theodorou, Triantafyllos Doskas, Evaggelos Fillopoulos

**Affiliations:** 1 Laboratory of Neuromuscular and Cardiovascular Study of Motion (LANECASM) Physiotherapy Department, Faculty of Health and Care Sciences, University of West Attica, Athens, GRC; 2 Medicine, 1st Vocational Lyceum, Volos, GRC; 3 Nursing, Larissa General Hospital, Larissa, GRC; 4 Health Care Management, School of Social Sciences, Hellenic Open University, Patras, GRC; 5 Department of Neurology, University Hospital of Alexandroupolis, Athens, GRC; 6 Department of Neurology, Athens Naval Hospital, Athens, GRC; 7 Oncology, Athens Euroclinic Breast Center, Athens, GRC

**Keywords:** multiple sclerosis, thalassemia, cancer, psychometric properties, fatigue

## Abstract

Introduction: Fatigue is associated with various diseases of different origins extending from immune disorders to cancer. The purpose of this study was to explore the psychometric properties/dimensionality of the Multidimensional Fatigue Inventory (MFI-20) questionnaire in samples of healthy adults and patient subgroups in Greece.

Methods: This was a multicenter cross-sectional study that included 80 women with breast cancer, 108 patients with multiple sclerosis (MS), 100 people with thalassemia diagnosis, and 185 healthy adults, aged 18-45 years. All patients were adults. Patients were recruited from a breast surgery clinic, a neurological clinic, and a thalassemia unit, while healthy adults were recruited from the University of West Attica students and personnel. The MFI-20, the modified fatigue impact scale (MFIS), the fatigue severity scale (FSS), and the Hamilton anxiety-depression scale (HANDS) were used. Internal consistency, repeatability, test-retest reliability, construct, and convergent validity were investigated.

Results: MFI-20 exhibited excellent reliability properties (internal consistency: Cronbach’s alpha MFI-20 subscales ranged from 0.83 to 0.94; repeatability: Pearson’s r = 0.335 [p < 0.001]). Significant correlations were found between MFI-20 and MFIS: Pearson’s r = 0.870, FSS: Pearson’s r = 0.582 - 0.335, and HANDS: Pearson’s r = 0.734 - 0.442 (all p-values < 0.0001) on all subsamples. However, its dimensionality is questionable depending on the subpopulation tested, and the one-dimension perspective is possible. MS patients exhibited the highest total score (55.26 ± 16.53), while thalassemia patients exhibited the lowest score (45.09 ± 13.54). In all subscales, thalassemia patients differed statistically significantly from the MS patients (p < 0.01), while in the reduced activity subscale, thalassemia patients differed significantly from all other groups (p < 0.01).

Conclusions: As strict fatigue subscale classification is questionable, the use of MFI-20 total score is suggested for the assessment of fatigue in clinical populations. As MFI-20 is a very useful research tool for studying fatigue, the use of the total and/or partial scores depends on the clinical population. Total score instead of (or additionally) partial scores is suggested in clinical practice.

## Introduction

Fatigue is a subjective, unpleasant, and multifactorial entity, which includes the embodiment of emotions ranging from tiredness to exhaustion, interfering with an individual's ability to cope with daily living activities. It is associated with various diseases of different origins extending from the immune system to cancer [1,2]. The subjectivity and the multidimensional nature of fatigue may be attributed to a synthesis of biological and psychological factors [3]. Many researchers approach fatigue from different points of view, and a variety of fatigue assessment tools have been developed. Some of them are recommended for specific diseases, and others can be used in more than one disease [4,5] and are categorized as one-dimensional or multidimensional. The conceptual interaction between dimensions remains unclear, which poses questions when interpreting the partial scores [6]. Some researchers even challenge the usefulness of the subscale scores implying that the total score, although not recommended by the manufacturer, would be more appropriate [7-11].

The multidimensional fatigue inventory (MFI-20) was developed to meet the need for a questionnaire that excludes somatic items (such as headaches) and measures multiple dimensions of fatigue [4]. It is a 20-item self-report questionnaire with five subscales (general fatigue, physical fatigue, reduced activity, reduced motivation, and mental fatigue) and has been widely used in numerous studies as a research instrument for a variety of diseases. Although it has been validated in many countries [11-18], its construction validity remains under question and there are serious concerns about cultural adaptations of the scale [11,12,15-18].

A previous attempt to validate the scale in Greece raised methodological problems and confirmed the aforementioned issues [19]. Regarding the MFI-20 Greek version and taking into account the previous research findings worldwide, we hypothesized that it would be necessary to further explore the structure of the questionnaire and apply the total score in addition to the partial scores suggested in the past.

## Materials and methods

This was a multicenter cross-sectional study that was conducted at the breast surgery clinic at the “Agios Savvas” Cancer Hospital of Athens, the Department of Neurology of Athens Naval Hospital, and the Mediterranean Anemia Unit of the General Hospital of Larissa (Central Greece) during 2018-2020. The study took place under the auspices of the Laboratory of Neuromuscular and Cardiovascular Study of Motion, University of West Attica.

Ethics

The research ethics committee of the Naval Hospital, the cancer hospital “Agios Savvas,” and the General Hospital of Larissa approved the study protocol. All the participants have signed and returned an informed consent conducted in accordance with the ethical principles stated in the Declaration of Helsinki and its later amendment.

Sampling

Four subpopulations were studied: women with breast cancer, multiple sclerosis (MS) patients, thalassemia patients (random sampling), and healthy adults (convenience sampling).

Research Sample No. 1

Research sample no. 1 consisted of 80 Greek adult women (above 18 years old) with breast cancer who were cancer survivors (a person who is living with and beyond a cancer diagnosis) [20], free of any other fatigue-causing disease, and randomly selected from the records of a breast surgery clinic of “Agios Savvas” Cancer Hospital of Athens.

The exclusion criteria were as follows: (a) women who did not complete their cancer treatment and (b) women who have completed their treatment recently, so they are not cancer survivors.

Research Sample No. 2

Research sample no. 2 consists of 108 Greek adult patients with MS who were treated in the regular outpatient clinics at the Department of Neurology of Naval Hospital (Athens). All patients were Greek with a definite diagnosis of MS, according to the revised McDonald’s criteria [21].

The exclusion criteria were as follows: (a) relapse less than one month before the assessment, (b) relapse between the two assessments, (c) coexisting disease, and (d) inability to visit the clinic, follow the instructions provided by the principal researcher, or respond to the questionnaires (expanded disability status scale [EDSS] < 7.0).

Research Sample No. 3

Research sample no. 3 consisted of 100 Greek adult patients with a diagnosis of thalassemia major, regardless of the severity of the disease. The research population resides in Thessaly and is monitored and transfused at regular intervals (every 15-20 days) in the Mediterranean Anaemia Unit of the General Hospital of Larissa (Central Greece). The patients have been transfusing for at least one year.

The exclusion criteria were as follows: patients who have been transfusing for a period of less than one year at the specific unit.

Study Sample No. 4

Study sample no. 4 consisted of 185 healthy adults, men and women, aged 18-45 years. Participants comprised students and employees of the University of West Attica. In 55 of the 185 subjects, the test-retest was performed at an interval of a week to avoid a learning effect.

The exclusion criteria were as follows: all participants were free of any fatigue-causing disease (e.g., asthma, thyroidism, etc., with no chronic disease and no medications for any reason during the previous months). Regarding study samples 1-3, questionnaires were completed in the presence of the researcher.

Procedure

In the present study, internal consistency, repeatability, test-retest reliability, construct, and convergent validity were investigated. For the purpose of the study, translation validity evidence of the MFI-20 was provided according to the standard recommended procedures. The group of patients and non-patients indicated that the translation of item numbers 9 and 19 needed further linguistic adaptations. After the appropriate modifications, questionnaires were administered first to cancer patients and then to MS patients and healthy individuals. Thalassemia patients were the last group to receive the MFI-20 questionnaire. Questionnaires were administered over a two-year period (2018-2020). MFI-20, modified fatigue impact scale (MFIS), fatigue severity scale (FSS), and Hamilton anxiety-depression scale (HANDS) were used to assess convergent validity. MFIS (the recommended fatigue scale for the MS population) [22] and HANDS were used in MS patients, FSS and HANDS were used in cancer patients, and FSS was used in healthy adults. All these scales have been validated in Greek populations. FSS and MFIS were validated by the same primary investigator [23,24]. HANDS has also been validated in Greek [25].

Instruments

The MFI-20 is a 20-item self-assessment questionnaire and was developed by Smets et al. in Dutch for cancer patients receiving radiation, in 1995 [4]. Respondents indicate the fatigue they have experienced lately. Respondents use a scale ranging from 1 to 5 to indicate how certain statements apply regarding fatigue to represent their experiences. Several positively phrased items are reverse scored. A higher total score is indicative of higher levels of fatigue. It is a five-point Likert scale (1 = yes, that is true to 5 = no, that is not true).

Permission to use the MFI-20 for the purpose of the present study was obtained from Smets et al. The FSS, one of the most widely used scales, is a nine-item self-assessment, unidimensional, rating scale [5]. For the purpose of the study, we used the validated Greek version of the FSS [23]. The MFIS, the recommended scale for the MS population, is a 21-item self-administered questionnaire [22]. For the purpose of the present study, we used the validated Greek version [24].

The HANDS is a self-report rating scale of 14 items on a four-point Likert scale (range: 0-3). It is designed to assess anxiety and depression (seven items for each subscale). The total score is the sum of the 14 items, and for each subscale, the score is the sum of the respective seven items (ranging from 0 to 21). For the purpose of the present study, we used the validated Greek version [25].

Statistical analysis

Statistics were performed with SPSS (Statistical Package for the Social Sciences) v22.0 software for windows (IBM Corp., Armonk, NY). Internal consistency was assessed by using Cronbach’s alpha. Principal component analysis was performed to examine the MFI-20’s factor structure. The convergent validity of the total score was tested using the Pearson correlation coefficients with the HANDS, MFIS, and FSS. For test-retest reliability, paired t-tests and scree plot diagrams were applied. Differences between the fatigue groups were tested using ANOVA (analysis of variance) and posthoc Bonferroni test. The significance was set at p = 0.05 level.

## Results

MFI-20 total and subscale scores (subscales as proposed by the manufacturer) of the four subpopulation groups along with basic demographic information are depicted in Table 1. MS patients exhibited the highest total score (55.26 ± 16.53), and thalassemia patients exhibited the lowest one (45.09 ± 13.54). In all subscales, a statistically significant difference was observed in the thalassemia patients than the MS patients (p < 0.01), while in reduced activity subscales, the thalassemia patients differed significantly from all other groups (p < 0.01) (Table 1).

**Table 1:** Demographic and comparative presentation of multidimensional fatigue inventory (MFI-20) subscale scores of each study group ANOVA, posthoc analysis: *Differences were significant for MS vs thalassemia patients (p < 0.01). **Thalassemia vs all other groups. The significance level of p-value < 0.05.

	Healthy adults (Greece), Ν = 185 (men/women: 91/94, aged 22.20 ± 3.02 years old)	MS patients, Ν = 108, (men/women: 37/70, aged 45.12 ± 12.30 years old)	Thalassemia patients, Ν = 100 (men/women: 43/57, aged 39.35 ± 8.52 years old)	Breast cancer patients, Ν = 80 (women, aged 48.32 ± 10.82 years old)	p-value
	Mean ± Std. deviation	Mean ± Std. deviation	Mean ± Std. deviation	Mean ± Std. deviation	
General/Physical fatigue	21.96 ± 5.35	25.18 ± 7.95	20.16 ± 7.08	21.45 ± 8.49	0.01*
Mental fatigue	9.83 ± 4.57	9.73 ± 4.10	7.67 ± 3.55	8.86 ± 3.15	0.01
Reduced activity	10.22 ± 3.58	10.93 ± 4.70	9.32 ± 3.65	9.51 ± 3.41	0.01**
Reduced motivation	8.47 ± 3.60	9.42 ± 4.05	8.03 ± 3.26	9.58 ± 3.86	0.01*
Total MFI score	50.51 ± 13.83	55.26 ± 16.53	45.09 ± 13.54	49.17 ± 17.86	0.01*

**Figure 1:**
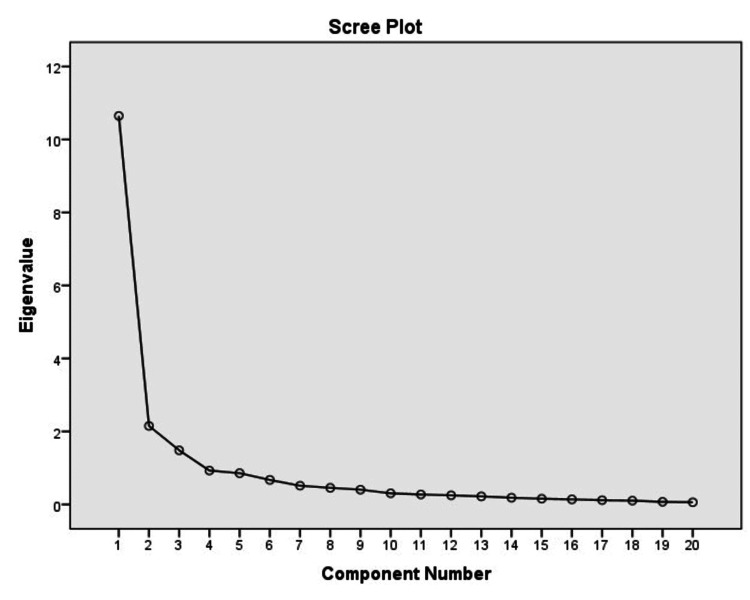
Eigenvalues scree plot before the rotation

Validity and reliability of MFI-20 were first explored in study sample 1. Data suitability for factor analysis was examined with the Kaiser-Meyer-Olkin (KMO) measure, which examines the partial correlations between the data, and its value should be greater than 0.60 to achieve a satisfactory analysis (it equals 0.89 in the present study). The survey data were first analyzed with the primary factor analysis method (principal component analysis [PCA]), with varimax rotation. The analysis resulted in three factors that explained 71.4% of the total variance. However, the eigenvalue graph clearly shows one factor, corresponding to the factor loadings table of the questionnaire data, before the rotation (Figure 1 and Table 2). Nevertheless, after rotation, three factors emerged, with the general and physical fatigue subscales being combined into one factor (although some elements of these subscales are found in the other two factors), while the elements from the other subscales are scattered in all three factors. Factor 1 (general fatigue/physical fatigue) explains 53.4% of the total variance and included all of the “general fatigue” and “physical fatigue” items, along with some other items.

**Table 2:** Factor analysis of the multidimensional fatigue inventory (MFI-20)

Item loadings across factors			
	Factor 1	Factor 2	Factor 3
x1	0.842 (0.372)		
x2	0.734 (0.674)		
x3	0.814	0.347 (0.805)	(0.318)
x4	0.767	0.349 (0.812)	
x5	0.785 (0.622)	(0.550)	
x6		0.543	0.528 (0.798)
x7	0.635	0.455 (0.353)	0.383 (0.767)
x8	0.507	(0.646)	-0.343
x9	0.675 (0.583)	(0.450)	
x10	0.575 (0.673)		0.433 (0.367)
x11	0.759 (0.460)	(0.361)	0.309 (0.606)
x12	0.770 (0.367)	(0.714)	
x13	0.676 (0.874)	-0.492	0.312
x14	0.882 (0.816)		
x15	0.733	0.461 (0.766)	
x16	0.840 (0.765)		
x17	0.815 (0.768)		
x18	0.809 (0.859)	-0.387	
x19	0.785 (0.711)	(0.349)	
x20	0.658 (0.319)	0.323 (0.304)	0.389 (0.703)

When factor analysis was applied to other study samples, four factors emerged finally for thalassemia patients, MS patients, and healthy people. In any case, factors were “hybrid” in the sense that comprised items belonging to different factors proposed by Smets et al. [4]. Regarding reliability (i.e., internal consistency), the Cronbach’s alpha coefficient ranged at high levels (0.83-0.94), the highest observed in breast cancer patients (total scale and general fatigue subscale), regardless of the factors retrieved (Table 3). Intraclass correlation value was 0.891 (p < 0.001).

**Table 3:** Internal consistency of multidimensional fatigue inventory (MFI-20) depending on the samples of the four studies MS: Multiple sclerosis.

Subscales Cronbach’s alpha	Healthy adults		MS patients		Breast cancer patients		Thalassemia patients	
General fatigue (items: 1, 5, 12, 16)	0.56	0.78	0.89	0.82	0.87	0.91	0.87	0.89
Physical fatigue (items: 2, 8, 14, 20)	0.74	0.74	0.74	0.75				
Reduced activity (items: 3, 6, 10, 17)	0.70		0.79		0.67		0.74	
Reduced motivation (items: 4, 9, 15, 18)	0.74		0.72		0.82		0.74	
Mental fatigue (items: 7, 11, 13, 19)	0.91		0.67		0,82		0.70	
Total fatigue	0.89		0.90		0,94		0.91	

**Figure 2:**
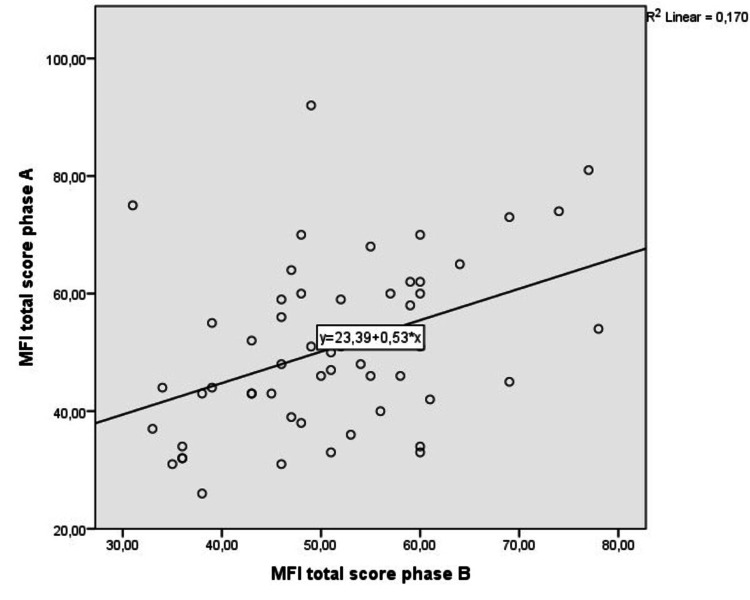
Scatter-plot diagram for multidimensional fatigue inventory (MFI-20) repeatability

When comparing MFI-20 to other well-established tests (concurrent validity), MFI-20 showed an excellent correlation against MFIS (MS patients, r = 0.870, p < 0.001), FSS (cancer group and healthy adults, p < 0.001), and HANDS (either depression or anxiety scale-healthy adults and MS patients, p < 0.0001). The fit line on the scatter plot diagram visualizes the linear relationship between the two measurements (Figure 2 and Table 4). MFI-20 also showed excellent repeatability and test-retest reliability in healthy individuals, while convergent validity was high, regardless of the population or the scales tested (intraclass correlation coefficient: 0.898, p < 0.001).

**Table 4:** Reliability and validity indexes of multidimensional fatigue inventory (MFI-20) *Cancer group, **Healthy adults, ***Multiple sclerosis patients. The significance level of p-value < 0.05. MFIS: Modified fatigue impact scale; FSS: Fatigue severity scale; HADS: hospital anxiety and depression scale.

Index	Test	Value		Significance (p)
Repeatability*	Pearson’s r	0.335		<0.001
Test-retest reliability*	Paired samples t-test	-0.173		0.863
Concurrent validity	Pearson’s r	Scale	r	
		MFIS	0.870	<0.001
		FSS	0.582** - 0.335*	<0.001
		HADS-depression	0.836** - 0.721***	<0.001
		HADS-anxiety	0.734** - 0.442***	<0.001

## Discussion

In the present study, we applied a new Greek version of the MFI-20 questionnaire after content and face validity procedures took place. To the best of our knowledge, this is the second study [8] comparatively examining the psychometric properties of the MFI-20 in multiple clinical populations that often display similar fatigue symptoms of dissimilar etiology [22,26,27]. In Greece, a previous effort to validate the MFI-20 questionnaire had been made by Aslani et al. [19] in a mixed Greek hemoglobinopathies population. However, the cross-cultural adaptation procedures were not fully described, and despite the encouraging reliability and convergent validity results, the factor analysis raised serious concerns about factor loading regarding MFI-20's physical fatigue and reduced activity subscale items, thus the validity of that version remains questionable [19].

The interesting finding of this study was the distribution variability of the items among the different subscales. There was no consensus regarding the number of factors emerging (the component analysis revealed three subscales for the women with breast cancer as opposed to four subscales for all other groups, including the healthy adults). Moreover, total scores rather than the subscale scores seem to be more appropriate in assessing fatigue in clinical settings. This deviation from the original structure proposed by Smets et al. was not actually a surprise, to us, since previous research has reported similar findings [8,13,15-18].

Specifically, for women with breast cancer, the three factors have not been classified according to Smets et al. [4] because the assignment of the items into the factors was too different and it was highly questionable. The first factor included 10 items in total comprising two items from each subscale (5, 16, 2, 14, 10, 7, 8, 18, 3, and 19). All the above items refer to tasks that people cannot “do,” i.e., express their inability with a potentially negative impact on their life. The second factor comprised six items (1, 3, 4, 8, 12, and 15) that express their positive feelings and aspects. The third factor incorporated four items (6, 7, 11, and 20) that are related to their positive attitude and their experience. It is worth mentioning that the first sample was a specific target group i.e., women with breast cancer who were free of cancer, in the fifth decade of their life with many family obligations and living under the threat of death like the sword of Damocles. According to De Raaf [28], physical fatigue and mental fatigue behave differently in cancer patients and the question is whether physical fatigue and mental fatigue are separate phenomena.

Probably, this particular female population experience fatigue in a unique way, given the multifaceted struggle in the biological and social fields of life. The three-factor model has been suggested by a German study with seven samples [8], which has included the cancer population.

The results of the MS group showed that the domains of general and physical fatigue were strongly correlated and fell under the same factor, which is in agreement with other researches [13,16]. The reduced activity factor included three items instead of four of the original scale because the item 3 (I feel very active) was conceived as more relevant to general/physical fatigue factor, by MS patients, probably due to the different semantic burden of the two words (feel and active), which in turn may be attributed to cultural factors. A similar finding had emerged in the factor analysis of the Greek version of MFIS [24]. The explanation for the different allocation of the items can be attributed to the way patients emphasize some words over others, due to cultural issues. The distribution of items of other subscales (mental and reduced motivation) was exactly as Smets et al. have proposed.

Regarding the factor analysis of thalassemia patients, the assignment of the components into the four dimensions has been quite differentiated from the MS sample, resampling the component structure in the healthy population. The first factor concluded all the items of general fatigue, two items from physical fatigue (2 and 20), and one item from reduced activity (3). The second factor incorporated five items (9, 13, 14, 17, and 18). Five items (4, 6, 8, 10, and 15) belonged to the third factor, which according to the original structure are two in reduced motivation (4 and 15), two in reduced activity (6 and 10), and one in physical fatigue (8). The fourth factor represented the dimension of mental fatigue because it includes all items of the original structure except for item 13 (it takes a lot of effort to concentrate on things).

The factor analysis results of the healthy population have shown four dimensions, and the allocation of the items into factors are assigned as follows: the first factor included the items from physical fatigue plus the item 1 from general fatigue, and the second one included the items (9, 16, 17, and 18) which according to Smets et al. are classified into reduced motivation (9 and 18), general fatigue (16), and reduced activity (17). In the third one, six items (3, 4-6, 12, and 15) have been incorporated; according to the original distribution, there were two items from reduced activity (3 and 6), two items from reduced motivation (4 and 15), and two items from general fatigue (5 and 12). The fourth factor represented the dimension of mental fatigue as it included all items of the original structure (7, 11, 13, and 19) plus the item from reduced activity (10).

Regarding MFI-20 reliability, Cronbach’s alpha coefficient was high (range: 0.83-0.94) for all subgroups, in line with some studies [8,16] despite the results of other studies showing modest reliability [7,13,18]. Our results were encouraging in terms of the concurrent validity and the repeatability/test-retest reliability and were in agreement with other studies [13]. MFI-20 showed an excellent concurrent validity when tested against MFIS, FSS, and HANDS. Repeatability and convergent validity were high for all groups in accordance with some but not all previous researches [7,15,16,18].

The interpretation of the results of the factor analysis in various clinical populations showed that the MFI-20 scale is a valid and reliable tool for screening fatigue; however, a different factor structure fits better in some groups than others, and it is in agreement with the German research in seven samples and the Swedish research on patients with post-polio syndrome [8,9]. The total score has also been proposed after the Rasch analysis model [9,10]. For instance, clinicians could use the four-factor structure of the MFI-20 for assessing fatigue in MS patients; however, a “hybrid” factor approach seems more appropriate for assessing fatigue in thalassemia and healthy adults. The clinicians’ dilemmas refer to the recommended scale for each disease, the number of the dimensions (a one-dimensional or multidimensional scale), and the number of items for each dimension. The unidimensional scales are easy to apply, but they usually focus on one specific dimension. The multidimensional scales approach more than one dimension but usually present problems with the validity of the structure, which result from the number of items for each dimension as well as from the interaction between items [8].

Differences in MFI-20 scores across the fatigue groups

Different qualitative fatigue characteristics of the various patient subpopulations may account for fatigue scores deviation between groups and the different item distribution among fatigue subscales. The finding that the MS patients exhibited the highest total and subscale scores was completely expected as it is the most frequent and disabling symptom of their disease.

Significant fluctuation of fatigue is not observed in patients with MS because they may avoid worsening fatigue caused by climatic conditions with the appropriate organization of their life routine [29]. Contrary to the MS group, the lower fatigue values in the thalassemia patients may be attributed to their good adaptation to the disease and the high scores they often exhibit in the social field of quality of life [30]. The close relationship between generalized fatigue and physical fatigue observed in MS patients could be a consequence of the patients’ difficulty origins. One possible explanation may be found in the subjective experience of fatigue. A person with an illness may experience dimensions of fatigue unknown to the healthy or may also be well adjusted to illness and express less discomfort. Besides that, seasonal variations of fatigue may also exert an influence on fatigue measurements [29]. The emotional component of fatigue refers to the general negative emotion that accompanies the subjective experience of fatigue. This dimension is related to the mental discomfort it may cause, and therefore, the latter can be used as a validity criterion for the scale under consideration. Anxiety and depression are often used to assess mental distress in cancer patients, and a high correlation was observed between the dimensions of HANDS and MFI-20, especially in the dimension of depression, a finding reported by other researchers [11]. Although this argues for the important emotional component of fatigue, it also raises some questions about the validity of the scale as there seems to be a significant overlap between fatigue and depression, especially a dimension of generalized fatigue and reduced motivation.

Strengths

The major strength is the suggestion of using a total score that resulted from the application in three different populations with different underlying causes of fatigue. Although the manufacturers do not recommend the use of the overall score, in the present study, treating the scale as unidimensional led to equally strong correlations with FSS and HANDS. As no mental disorders were present in the sample's contribution, psychiatric morbidity in the results was minimized.

The rather “hybrid” construction of the factors in the present study is probably indicative of a subjective experience of fatigue, which could also change over time. This hybrid construction finding had been also reported in the Canadian version of the scale, where the interpretation of variance reached 41% for the first factor [11]. In the same study, it was noted that no control-retest was performed, mainly because fatigue shows high variability over time. It could be difficult to obtain reliable results from two measurements of a person's experience of fatigue in a short time.

Limitations

The primary limitation to the generalization of these results is the selection of the specific subpopulations and the lack of controlling other psychological factors that may influence the self-reported fatigue experience, i.e., psychiatric comorbidities. The second limitation is the period of the conduct of the study (2018-2020), a period of financial and social difficulties in Greece because the country was under a finance memorandum. It can explain the feeling of fatigue experienced by the subject under circumstances of discomfort.

## Conclusions

Fatigue is a multidimensional phenomenon, and each patient subpopulation exhibits unique fatigue characteristics. As people with different underlying diseases experience fatigue in different ways, a single tool, no matter how multidimensional it may be, cannot be suitable for all subpopulations. The study showed that the MFI-20 total score (of the new Greek version) is a valid and reliable index of fatigue, regardless of the clinical population. The total score instead of (or additionally) partial scores is suggested for clinical practice. However, when there is a necessity for a multidimensional approach, the clinician should explore the dimensions suggested for each clinical population, according to published data.
